# Fidelity of classwide-resistant HIV-2 reverse transcriptase and differential contribution of K65R to the accuracy of HIV-1 and HIV-2 reverse transcriptases

**DOI:** 10.1038/srep44834

**Published:** 2017-03-23

**Authors:** Mar Álvarez, Alba Sebastián-Martín, Guillermo García-Marquina, Luis Menéndez-Arias

**Affiliations:** 1 Centro de Biología Molecular “Severo Ochoa” (Consejo Superior de Investigaciones Científicas and Universidad Autónoma de Madrid), Madrid, Spain

## Abstract

Nucleoside reverse transcriptase (RT) inhibitors constitute the backbone of current therapies against human immunodeficiency virus type 1 and type 2 (HIV-1 and HIV-2, respectively). However, mutational pathways leading to the development of nucleoside analogue resistance are different in both types of HIV. In HIV-2, resistance to all approved nucleoside analogues is conferred by the combination of RT substitutions K65R, Q151M and M184V. Nucleotide incorporation kinetic analyses of mutant and wild-type (WT) HIV-2 RTs show that the triple-mutant has decreased catalytic efficiency due to the presence of M184V. Although similar effects were previously reported for equivalent mutations in HIV-1 RT, the HIV-2 enzymes were catalytically less efficient. Interestingly, in highly divergent HIV-1 RTs, K65R confers several-fold increased accuracy of DNA synthesis. We have determined the intrinsic fidelity of DNA synthesis of WT HIV-2 RT and mutants K65R and K65R/Q151M/M184V. Our results show that those changes in HIV-2 RT have a relatively small impact on nucleotide selectivity. Furthermore, we found that there were less than two-fold differences in error rates obtained with forward mutation assays using mutant and WT HIV-2 RTs. A different conformation of the β3-β4 hairpin loop in HIV-1 and HIV-2 RTs could probably explain the differential effects of K65R.

Human immunodeficiency viruses type 1 and type 2 (HIV-1 and HIV-2, respectively) share many traits, including similar genetic organizations, major intracellular replication pathways, mode of transmission, and clinical effects leading to the acquired immunodeficiency syndrome (AIDS)^1^. However, HIV-2 is less pathogenic, undergoes a longer asymptomatic phase and shows lower rates of transmission^2^,^3^. Despite the slower progression to AIDS, many HIV-2-infected patients develop clinical features indistinguishable from the syndrome caused by HIV-1, while having fewer treatment options available. Attempts to correlate mutation frequencies in HIV-1 and HIV-2 with the attenuated progression to AIDS have been inconclusive^4^,^5^,^6^. Several factors including dNTP pools, viral proteins and cell types may have an important effect on the viral mutation rate. Next-generation sequencing analysis of amplicons obtained from infected cells showed that HIV-2 had lower mutation rates than HIV-1, although the observed differences were due to the high frequency of G → A transitions found in HIV-1^7^. The higher susceptibility of this virus to APOBEC3-mediated hypermutation could explain these results^8^. Nucleotide incorporation assays carried out with heteropolymeric template-primers under steady-state conditions revealed only subtle differences in misinsertion and mispair extension fidelity between HIV-1_BH10_ and HIV-2_ROD_ reverse transcriptases (RTs)^9^. However, a proper assessment of the intrinsic fidelity of the HIV-2 RT, including estimates of base substitution and frameshift error rates relative to other RTs is still missing.

HIV-1_BH10_ and HIV-2_ROD_ RTs share similar DNA polymerase catalytic properties^10^,^11^. However, the HIV-2_ROD_ enzyme has reduced processivity, particularly in the presence of low dNTP concentrations^12^. In addition, Lenzi *et al*. have recently shown that RTs of several HIV-2 and simian immunodeficiency virus (SIV) strains require higher dNTP concentrations for efficient DNA synthesis^13^. These differences would be most relevant when considering viral replication kinetics in macrophages and non-dividing cells that harbour very low dNTP concentrations. In myeloid cells, expression of the host factor SAMHD1 (sterile α motif and HD domain-containing protein-1) restricts HIV replication by reducing the intracellular dNTP pools, due to its deoxynucleoside triphosphate triphosphohydrolase (dNTPase) activity^14^,^15^,^16^,^17^. However, viral proteins such as Vpx (in HIV-2 and in SIV_smm_- and SIV_rcm_-related viruses) and certain variants of Vpr (e.g. those found in SIV strains from African green monkeys) counteract the effects of SAMHD1 by targeting the protein for proteasomal degradation^18^,^19^. Interestingly, Vpx-mediated degradation of SAMHD1 decreases HIV susceptibility to zidovudine (3′-azido-3′-deoxythymidine (AZT)) and stavudine (2′,3′-didehydro-2′,3′-dideoxythymidine (d4T)) in macrophages and lymphocytes^20^. Those findings were consistent with data from a previous report showing that purified SAMHD1 has significantly lower dNTPase activity against AZT-triphosphate than against natural dNTPs^21^. A link between the properties of RTs and Vpx/Vpr proteins has been suggested from phylogenetic analysis of their coding sequences in HIV and SIV genomes^22^.

HIV-2 shows natural resistance to a number of antiretroviral drugs designed to suppress HIV-1 propagation. Thus, nonnucleoside RT inhibitors (NNRTIs), the fusion inhibitor enfuvirtide and several protease inhibitors are ineffective against HIV-2, while the clinical efficacy of maraviroc is uncertain^23^. Unlike HIV-1, HIV-2 does not develop resistance to nucleoside RT inhibitors (NRTIs) through the excision pathway (involving amino acid substitutions M41L, D67N, K70R, L210W, T215F/Y and K219E/Q in the viral RT^24^), but relies exclusively on nucleotide discrimination^12^. However, clinical and epidemiological studies have shown that HIV-2 has lower genetic barriers than HIV-1 to the development of multidrug resistance^25^. NRTIs (usually tenofovir/emtricitabine) constitute the backbone of commonly prescribed therapies against HIV-2^23^,^26^. RT substitutions K65R and Q151M are frequently identified in virus obtained from treated HIV-2-infected patients^27^, and the presence of both changes together with M184V confers classwide NRTI resistance^28^. An estimated prevalence of 9% has been reported for the combination of K65R, Q151M and M184V in a European cohort of HIV-2-infected patients treated with antiretroviral drugs^29^.

Phenotypic studies using recombinant HIV-2 have shown that Q151M alone confers high-level resistance to AZT (zidovudine), and moderate- to low-level resistance to stavudine, abacavir, didanosine and emtricitabine. However, it has a small impact on the viral susceptibility to lamivudine and tenofovir^28^,^29^,^30^,^31^. As previously shown in HIV-1^24^, K65R arises in HIV-2-infected patients exposed to tenofovir and other NRTIs^27^,^32^, while M184V confers high-level resistance to approved cytidine analogues (i.e. lamivudine and emtricitabine)^28^. K65R can be selected *in vitro* after HIV-2 exposure to increasing doses of tenofovir^33^ and produces a 2- to 7-fold decrease in viral susceptibility to the drug^29^,^33^. In addition, K65R confers low-level resistance to didanosine and moderate to high-level resistance to lamivudine and emtricitabine in HIV-2 strains^28^,^30^. Lys^65^, Gln^151^ and Met^184^ locate at the dNTP binding site of the RT^34^. By themselves, amino acid substitutions K65R, Q151M or M184V in HIV-1 RT confer reduced catalytic efficiency of nucleotide analogue incorporation (review^24^). Consistently with those findings, mutant HIV-1 with RT substitutions K65R or M184V show delayed replication kinetics in comparison with the wild-type (WT) virus^35^,^36^. In the case of M184V, the fitness defect is more pronounced at low dNTP concentrations and is expected to be more relevant for viruses replicating in non-dividing cells (e.g. macrophages)^37^. HIV-1 RTs with Arg^65^ instead of Lys showed decreased nucleotide incorporation rates (*k*_pol_) in pre-steady-state kinetic assays^38^,^39^,^40^. Other studies have shown that K65R produces a >8-fold increase in the intrinsic fidelity of the HIV-1 RT, an effect that has been demonstrated with RTs of highly divergent HIV-1 group M (subtype B) and group O strains^40^,^41^. In agreement with enzymatic data, K65R produced a 3.3-fold mutant frequency reduction in single round of replication assays, using an HIV-1_NL43_ vector containing the *lacZ* gene^42^.

In contrast to the well-studied HIV-1 RT, studies on the effects of NRTI resistance-associated mutations on the nucleotide specificity and fidelity of the HIV-2 polymerase are limited to a few single- and double-mutants with substitutions at positions 74, 89, 111 and 215 (i.e. L74V, E89G, V111I, S215Y, L74V/S215Y and E89G/S215Y), whose effects were evaluated for a small subset of misincorporations or mispairs using nucleotide discrimination assays^29^,^43^. Unfortunately, those studies could not provide reliable mechanistic interpretations of the data because assays were performed under steady-state conditions. None of the studied residues were part of the complex conferring multi-NRTI resistance, although sometimes V111I appears as an accessory mutation that compensates for the lower fitness of classwide NRTI-resistant HIV-2^29^. In our study, we have examined the effects of K65R, Q151M and M184V on nucleotide incorporation, using pre-steady-state kinetics. We show that WT and mutant HIV-2 RTs have reduced nucleotide binding affinities in comparison with the equivalent HIV-1 enzymes, while M184V was found to be responsible for the reduced catalytic efficiency of the triple mutant K65R/Q151M/M184V. Unexpectedly, and unlike in the case of HIV-1 RTs, K65R had a relatively small impact on misinsertion and mispair extension ratios suggesting only subtle effects on the fidelity of HIV-2 RT. Furthermore, the error rates of mutant RTs K65R and K65R/Q151M/M184V were similar to those obtained with the WT HIV-2 RT, using an M13mp2 *lacZα* forward mutation assay. Although the RT mutation K65R seems to be selected in HIV-1 and HIV-2 after exposure to tenofovir and other NRTIs, our results suggest that there is limited functional equivalence between Lys^65^ in both HIV RTs.

## Results

### Nucleotide incorporation kinetics

**Figure 1:**
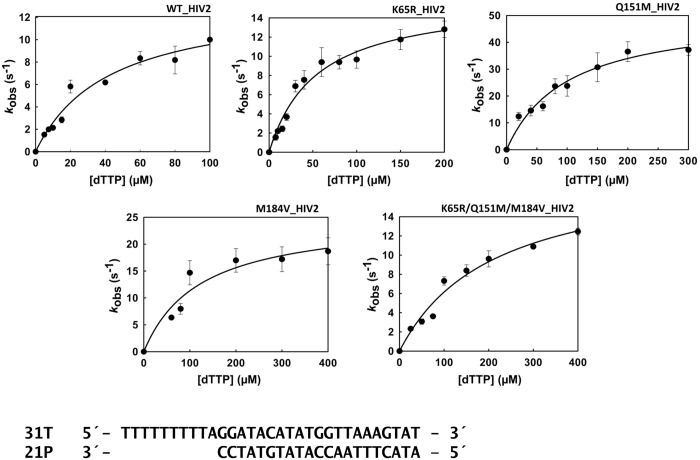
Nucleotide concentration dependence for the incorporation of dTTP into the 31T/21P duplex DNA for wild-type (WT) and mutant HIV-2 RTs. The continuous line represents the best fit of the data to the Michaelis-Menten equation.

The catalytic efficiency of nucleotide incorporation was determined for the WT HIV-2_ROD_ RT and mutants K65R, Q151M, M184V and K65R/Q151M/M184V under pre-steady-state conditions, using a 31/21mer heteropolymeric template-primer. Catalytic rates and nucleotide affinity constants (*k*_pol_ and *K*_d_, respectively) were obtained after representing nucleotide incorporation rates (*k*_obs_) *versus* dNTP concentrations and fitting the data to a hyperbola (Fig. 1). In these assays, the WT HIV-2 RT showed 3.5 times higher catalytic efficiency than the triple mutant K65R/Q151M/M184V (Table 1). These differences could be attributed to the higher *K*_d_ of the triple mutant. The amino acid substitution M184V alone produced a 3.9-fold reduction of the dNTP binding affinity and was found to be responsible for the lower catalytic efficiency of the K65R/Q151M/M184V HIV-2 RT. In contrast, K65R had minor effects on the catalytic constants determined in these assays, while Q151M produced a modest increase in the catalytic efficiency of the HIV-2 RT, despite reducing the nucleotide binding affinity by two-fold. Interestingly, in comparison with WT HIV-1 RTs of group M-subtype B (BH10 strain)^44^ or group O (ESP49 strain)^40^, the HIV-2 enzymes showed 2.6- to 3.9-fold reduced catalytic efficiencies, as a consequence of their lower affinity for incoming dNTPs.

### Pre-steady-state kinetic analysis of fidelity of DNA synthesis of WT and mutant HIV-2 RTs

**Figure 2:**
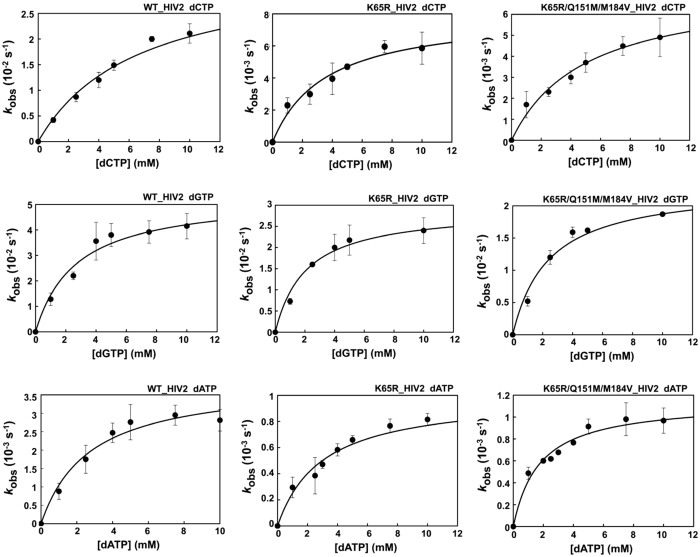
Nucleotide concentration dependence on the first-order rates for dCTP, dGTP and dATP incorporation into the DNA/DNA 31/21-mer 31T/21P, obtained with WT and the mutant HIV-2 RTs K65R and K65R/Q151M/M184V. The first-order rates of the nucleotide incorporation reaction (*k*_obs_) were plotted against the corresponding nucleotide concentration. The data were fitted to the Michaelis-Menten equation to obtain *K*_d_ and *k*_pol_ values (best fit represented with a solid line).

Previous studies with prototypic HIV-1 RTs had shown that K65R produced large increases in the fidelity of DNA synthesis in the absence^40^,^41^ and in the presence of the NRTI-associated resistance mutation V75I^40^. We analysed the nucleotide specificity of HIV-2_ROD_ RT mutants K65R and K65R/Q151M/M184V in misinsertion and mispair extension fidelity assays. The kinetics of misincorporation of dCTP, dGTP and dATP at the 3′ end of the primer into the 31T/21P heteroduplex were determined under single-turnover conditions for mutant and WT RTs. The obtained incorporation rates (*k*_obs_) were represented *versus* dNTP concentrations, and the data fitted to the Michaelis-Menten equation (Fig. 2). The obtained *k*_pol_ and *K*_d_ values, and the catalytic efficiencies (*k*_pol_/*K*_d_) of nucleotide misincorporation are given in Table 2. The highest catalytic efficiencies were observed for the incorporation of non-complementary dCTP (1.5 × 10^−6^–5.2 × 10^−6^) and dGTP (9.6 × 10^−6^–2.0 × 10^−5^), and were about four to five orders of magnitude lower than those obtained for the correct nucleotide. Large reductions in the catalytic efficiencies of incorporation of C, G or A opposite A result from their lower *k*_pol_ and the large increases in the *K*_d_ values obtained in comparison with the corresponding parameters determined for the correct nucleotide (i.e. dTTP). Differences in the misinsertion ratios between the WT HIV-2_ROD_ RT and mutants K65R and K65R/Q151M/M184V were small and non-significant except for the misincorporation of A opposite A which was about 4 times less efficient for the single-mutant K65R than for the WT RT. Nevertheless, dATP incorporation is very inefficient in this sequence context, with *k*_pol_/*K*_d_ values 6.5 and 44 times lower than those obtained with dCTP or dGTP, respectively, suggesting that this type of errors would be rare during reverse transcription.

**Figure 3:**
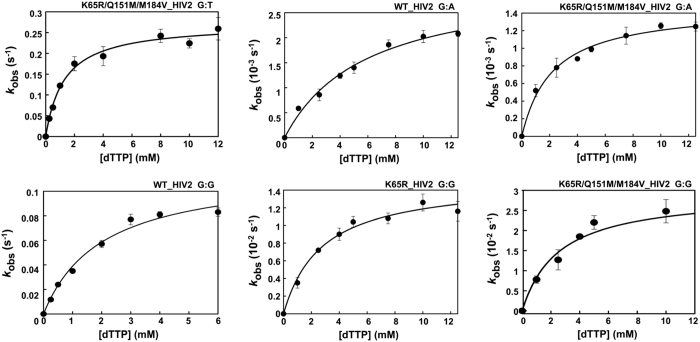
Nucleotide concentration dependence on the first-order rates for dTTP incorporation into DNA/DNA 31/21-mers with G:T, G:A and G:G mismatches at their 3′ end. The first-order rates of the incorporation reaction (*k*_obs_), obtained with WT and mutant HIV-2 RTs (K65R and K65R/Q151M/M184V), were plotted against the corresponding nucleotide concentration. The data were fitted to the Michaelis-Menten equation to obtain *K*_d_ and *k*_pol_ values (best fit represented with a solid line).

Previous studies with WT and mutant HIV-1 RTs have shown that mispair extension fidelity assays are more reliable for detecting differences in the accuracy of those enzymes^40^,^44^. We determined kinetic parameters for the incorporation of dTTP using template-primers 31T/21PT, 31T/21PG, and 31T/21PA. These complexes were identical to 31T/21P but contained mismatched 3′ ends (i.e. G:T, G:G and G:A in duplexes 31T/21PT, 31T/21PG, and 31T/21PA, respectively). The kinetic constants obtained for mispair extension reactions catalysed by WT and mutant HIV-2 RTs are shown in Table 3. As expected, G:T mismatches were extended more efficiently than G:G or G:A mispairs. However, the *k*_pol_ of the nucleotide incorporation reaction on G:T mismatches could not be reliably estimated for the WT and the mutant K65R RT, because both enzymes showed extremely low dTTP binding affinity in this sequence context. The same difficulties were also found for the K65R HIV-2 RT in G:A mispair extension assays. Plots of nucleotide incorporation rates *versus* dTTP concentrations are given in Fig. 3 for those mispairs providing valid estimates of *k*_pol_ and *K*_d_. Nucleotide incorporation rates (*k*_pol_) for the extension of G:T, G:G and G:A mispairs were very small except for reactions catalysed by the WT RT using G:T mismatched template-primers. However, where kinetic parameters were reliably determined, the differences between WT HIV-2 RT and the classwide NRTI-resistant K65R/Q151M/M184V RT were not significant. The K65R mutant enzyme showed increased fidelity in comparison with the WT RT in extension reactions with template-primers having a G:G mismatch at their 3′ ends. However, in this case the catalytic efficiency of nucleotide incorporation was very low (5.4 × 10^−6^ μM^−1^ s^−1^).

### M13mp2 *lacZα* forward mutation assays

Nucleotide incorporation assays detected only small differences of fidelity between WT and mutant HIV-2 RTs. However, they provided only limited information on nucleotide specificity since the analyses were restricted to a reduced number of substrates and sequence contexts. M13-based forward mutation assays using *lacZα* as a reporter gene provide a better fidelity assessment, based on a relatively large number of mutational sites, although silent mutations could not be detected using this method^45^. These assays were used to determine mutation frequencies and error rates for WT HIV-1_BH10_ and HIV-2_ROD_ RTs, as well as for mutant HIV-2 RTs K65R and K65R/Q151M/M184V. A double-stranded DNA derived from phage M13mp2 and lacking the *lacZα* gene sequence in one of the two DNA chains (gapped DNA) was used as substrate. Mutations introduced when the RT copied the gapped region of the *lacZ* gene changed the colour phenotype of M13mp2 plaques, detected when phages were grown on an appropriate indicator strain, in the presence of isopropyl β-D-1-thiogalactopyranoside (IPTG) and 5-bromo-4-chloro-3-indolyl-β-D-galactopyranoside (X-Gal). Mutant frequencies were calculated as the ratio of mutant (pale blue or colourless) to total plaques. In these assays we found that the WT RT of the HIV-1_BH10_ strain had lower accuracy than the WT HIV-2_ROD_ RT, although differences were relatively small with mutation frequencies of 1.99% and 1.24%, respectively (Table 4). Mutations K65R and K65R/Q151M/M184V had almost no effect on the intrinsic accuracy of the HIV-2_ROD_ RT, with mutant frequencies being only slightly lower in comparison with that of the WT enzyme.

The mutational specificity of the studied RTs was determined after sequencing the *lacZα* mutants generated in the forward mutation assays. Analysis of the obtained mutational spectra revealed small differences between WT and mutant HIV-2 RTs (Supplementary Figs S1–S4). Nevertheless, a few general trends were common to those three enzymes. For example, more than one third of all errors introduced by HIV-2 RT variants (mutant and WT enzymes) were frameshifts, and in most cases consisted of deletions of one nucleotide. These deletions occurred predominantly at heteropolymeric sequences. Base substitutions were frequently observed in all spectra, with transversions appearing at least three times more frequently than transitions in the analysed *lacZα* sequence. A predominance of G-to-T substitutions was detected in all mutational spectra. The analysis of error rates (Table 5) demonstrated that all HIV-2 RTs introduced frameshifts with a similar frequency, while differences in fidelity were observed when looking at base substitution error rates. Thus, the frequency of this type of mutations was reduced by less than two-fold in the case of classwide-resistant K65R/Q151M/M184V RT relative to the WT enzyme, while K65R alone produced a mere 1.5-fold increase in nucleotide substitution fidelity. The comparison of WT HIV-2_ROD_ and HIV-1_BH10_ RTs demonstrated that both enzymes introduced similar types of errors (i.e. frequent one-nucleotide deletions, and more transversions than transitions). However, frameshifts were more abundant in the mutational spectrum of the HIV-2_ROD_ RT (Supplementary Fig. S4). Compared with the WT HIV-1_BH10_ RT, the HIV-2 enzymes were less prone to introduce single nucleotide substitutions, but showed higher overall frameshift error rates due to their strong tendency to introduce deletions at heteropolymeric sequences.

## Discussion

Classwide NRTI resistance in HIV-2 is conferred by mutations K65R, Q151M and M184V^28^. In agreement with previous reports^13^,^46^, we found that HIV-2_ROD_ RT exhibits lower catalytic efficiency than HIV-1 RTs. Previously reported dNTP incorporation rates (*k*_pol_) for HIV-1 group M subtype B RTs (e.g. HIV-1_BH10_ and HIV-1_NL43_ RTs) were in the range of 11.6–17.4 s^−1^, with *K*_d_ values between 7.1 and 17 μM^44^,^47^,^48^, as determined in assays carried out with the same template-primer used in our study (i.e. 31T/21P). Those kinetic parameters were similar to those previously obtained for WT HIV-1_ESP49_ group O RT (*k*_pol_ = 13.2 s^−1^ and *K*_d_ = 17.3 μM)^49^. Under the same conditions, the HIV-2 RT shows similar incorporation rates but reduced nucleotide affinity, thereby suggesting a possible mechanistic explanation for the lower replication capacity of HIV-2 in different cell types^50^,^51^.

Mutations selected under drug pressure frequently result in a loss of viral fitness. Our analysis on the effects of K65R, Q151M and M184V on nucleotide incorporation kinetics revealed that M184V was responsible for the reduced catalytic efficiency of classwide-resistant HIV-2 RT. M184V alone decreases the catalytic efficiency by reducing the nucleotide binding affinity of the RT. Although K65R, Q151M and M184V might be selected under NRTI pressure in HIV-1 as well as in HIV-2, the classwide-resistant triple mutant is characteristic of the HIV-2 RT since Q151M is the preferred mutation selected during treatment with AZT^23^,^24^. Interestingly, the effects of those mutations on nucleotide incorporation by HIV-1 RTs had been previously assessed with the 31T/21P duplex, using pre-steady-state kinetics^38^,^39^. The results of those studies were consistent with the kinetic data reported here and showed that M184V produced a significant reduction of the catalytic efficiency of the HIV-1 polymerase due to a decreased dNTP binding affinity. In contrast, the substitution of Met for Gln^151^ (Q151M) enhanced the catalytic efficiency of the HIV-1 RT due to an increased *k*_pol_^38^, an effect that we also observed with this amino acid substitution in the HIV-2 RT. On the other hand, the results with K65R were slightly different in the HIV-1_BH10_ RT as compared with the HIV-2_ROD_ enzyme. Deval *et al*.^38^ showed that K65R reduced the catalytic efficiency of HIV-1_BH10_ RT by decreasing its nucleotide incorporation rates (*k*_pol_) for different dNTPs, while in our assays with HIV-2 RT, the substitution had almost no effect on the catalytic parameters of the enzyme. As in HIV-1_BH10_ RT, mutant HIV-1 group O RT bearing the K65R change showed reduced *k*_pol_, although in this case the enzyme had increased affinity for the incoming dNTP^40^.

In studies carried out with HIV-1 group O RT, K65R was found to have a large impact on nucleotide selectivity. Introduction of K65R in the sequence of HIV-1_ESP49_ RT decreased misinsertion and mispair extension ratios for all tested nucleotides and mismatched template-primers^40^. Furthermore, studies with the group O enzyme also showed the increased nucleotide selectivity of the double-mutant K65R/V75I in comparison with the RT bearing the single amino acid substitution V75I. In general, the results of our misinsertion and mispair extension fidelity assays using the 31/21-mer template-primer did not reveal significant differences between HIV-2 RTs bearing mutation K65R and the WT enzyme, except for the extension of G:G mispairs which was less efficient in the case of the mutant enzyme. Unfortunately, data were inconclusive for most mispairs, including the ones that were extended more efficiently, due to the low efficiency of dNTP incorporation observed with the used template-primers.

To overcome those limitations, we obtained more reliable estimates of the intrinsic fidelity of HIV-2 RTs by using forward mutation assays. It is well-known that lentiviral RTs (e.g. those of HIV-1, SIV or feline immunodeficiency virus) are less accurate than oncoretroviral RTs, such as those of murine leukaemia virus (MLV) or avian myeloblastosis virus^52^,^53^. Our study shows that the WT HIV-2_ROD_ RT is only 1.6 times more faithful than the reference HIV-1_BH10_ RT. These differences are similar to those reported for other primate lentiviral RTs when compared with HIV-1 group M subtype B polymerases, such as the RTs of HIV-1 strains BH10 or HXB2, using the M13-based forward mutation assay. Thus, the HIV-1_BH10_ RT showed about two-fold decreased fidelity in comparison with a prototypic WT HIV-1 group O RT^54^,^55^, while SIV_mne_ and SIV_agm_ RTs were only 1.3 and 1.8 times more accurate, respectively, than HIV-1 group M subtype B RTs^56^,^57^. Despite the relatively small differences in mutation rates, HIV-1 group M subtype B RTs emerge as the less faithful polymerases among those of primate lentiviruses. The higher intrinsic fidelity of HIV-2 RT in comparison with HIV-1_BH10_ RT results from its lower nucleotide substitution error rate, a trend that was also observed with the SIV_agm_ RT^56^. In contrast, our analysis shows that WT HIV-2_ROD_ RT has a relatively high frameshift error rate (5.7 × 10^−5^), ranking among the highest reported for RTs of primate lentiviruses.

There were relatively small differences in fidelity between the classwide NRTI-resistant K65R/Q151M/M184V RT and the WT HIV-2_ROD_ enzyme, despite the presence of K65R in the mutational complex. These effects were unexpected considering that K65R alone produced large increases in fidelity in forward mutation assays carried out with HIV-1 RTs from phylogenetically distant strains such as HXB2 (group M subtype B)^41^ or ESP49 (group O)^40^. Furthermore, estimates of HIV-1 mutant frequencies obtained in cell culture after one round of replication were consistent with the enzymatic data and showed a 3.3-fold reduction for virus containing the K65R mutation in their RT^42^. In contrast, our results show that K65R in HIV-2_ROD_ RT had almost no effect on the overall fidelity of the enzyme, although it produced a modest decrease of its base substitution error rate. The effects of K65R on the evolutionary rate of HIV-2 have not been assessed. However, a recent work has shown the quick reversion of the mutation when present in SIV_mac239_ infecting pigtailed macaques^58^. Interestingly, WT and mutant SIV_mac239_ showed similar mutation rates in cell-based assays although K65R-containing viruses showed slightly reduced variability. The differences in mutation rates were estimated to be less than 1.3-fold, but considered statistically significant^58^. Interestingly, mutant MLV carrying the equivalent substitution in its RT (i.e. K103R) was found to be less infectious than the WT. However, no differences were found in assays measuring the frequency of *lacZ* inactivation after one round of replication in cells infected with WT and mutant viruses^59^, thereby suggesting that the mutation had no effect on the intrinsic fidelity of the MLV RT.

Although several studies carried out with HIV-1 RT variants have shown that K65R and other mutations conferring resistance to nucleoside analogues (e.g. M184V) increase their DNA polymerase fidelity, there are examples of NRTI resistance mutations having little effect on the accuracy of HIV-1 RT (e.g. L74V, Q151M)^52^,^53^. Our results show that in HIV-2, classwide NRTI resistance may not be associated with significant changes in the intrinsic fidelity of its RT, although a lower fitness is expected for drug-resistant viruses as a result of the lower catalytic efficiency of their RT. Therefore, the relationship between drug resistance and increased fidelity cannot be established, particularly for the major mutations found in HIV-2 strains resistant to NRTIs.

The increased intrinsic fidelity conferred by K65R appears to be a specific characteristic of HIV-1 RT and not shared by RTs of the HIV-2/SIV lineage. Although a structural justification of these differences is very difficult due to the lack of structural information for HIV-2 RT, a number of amino acid sequence differences found in the β3-β4 hairpin loops (residues 63–75) of RTs of HIV-1 and HIV-2 could have a significant impact on its conformation and mobility, thereby affecting neighbouring contacts between the side-chains of Lys or Arg^65^. In support of this notion, crystal structures of ternary complexes composed of mutant K65R HIV-1_BH10_ RT, double-stranded DNA and incoming nucleotides (i.e. dATP or tenofovir-diphosphate) have shown stacking interactions between guanidinium groups of Arg^65^ and Arg^72^ that restrict the conformational adaptability of both residues^60^. The amino acid sequences of the β3-β4 hairpin loops of HIV-1 group M subtype B and group O RTs are identical (IKKKDSTKWRKLV), but different from those found in HIV-2_ROD_ and SIV_mac239_ RTs (IKKKDKNKWRMLI). The distribution of charged residues along the hairpin loop sequence is rather different in both pairs of RTs and it is tempting to speculate that substitutions of basic residues in these structures could have an impact on fidelity. Nevertheless, how interactions between hairpin loop residues may influence the intrinsic fidelity of the enzyme is a question open for future research.

## Experimental Procedures

### Expression and purification of RTs

WT heterodimeric (p66/p51) HIV-1_BH10_ RT was obtained as previously described^61^,^62^. Histidine-tagged WT and mutant HIV-2_ROD_ RTs were obtained as p68/p54 heterodimers with constructs derived from plasmid pT5m that were used to transform *Escherichia coli* strain BL21(DE3)(pLysS). The pT5m vector containing the p68-coding region of the WT HIV-2_ROD_ RT and the HIV-2_D194_ protease has been previously described^63^ and was kindly provided by Amnon Hizi (Tel Aviv University, Israel). A modified version of the method described by Boyer *et al*.^12^ was used to purify HIV-2 RTs. Briefly, bacteria (in one-liter Luria-Broth cultures, containing 2 mM glucose) were grown at 30 °C and RT expression was induced for 3 h with 1 mM IPTG. Cells were collected by centrifugation and the obtained pellets were re-suspended in 6 ml of 50 mM sodium phosphate pH 8.0 buffer, containing 50 mM NaCl, 0.75 mg/ml hen egg-white lysozyme and 1.5 mM phenylmethylsulfonyl fluoride (PMSF), and kept on ice for 25 min. After addition of 0.43 ml of 4 M NaCl, the solution was subjected to brief periods of sonication, and then centrifuged at 23,000 rpm for 60 min at 4 °C using a Beckman JA 25.50 rotor. The obtained supernatants were diluted with one volume of 66 mM sodium phosphate pH 7.0 buffer, containing 0.3 M NaCl, and applied on a 3 ml Ni^2+^-nitriloacetic acid-agarose column (Invitrogen, Carlsbad, CA, USA), previously equilibrated with 50 mM sodium phosphate pH 7.0 buffer containing 0.3 M NaCl. The column was then washed with 50 mM sodium phosphate pH 6.0 buffer containing 0.3 M NaCl, 20 mM imidazole and 10% glycerol. The RT was eluted with a 20 mM–0.5 M imidazole gradient in the same buffer. The fractions containing the heterodimer were analysed by SDS-PAGE, pooled and dialyzed against 50 mM sodium phosphate pH 6.8 buffer, and then applied on a 5 ml P11 phosphocellulose column (Whatman, Maidstone, Kent, England), previously equilibrated with the same buffer. After washing the column with 50 mM sodium phosphate pH 6.8 buffer containing 0.3 M NaCl, the RT was eluted with a 30-ml gradient from 0.3 to 1 M NaCl. Fractions eluted from the salt gradient were analysed by SDS-PAGE, and those containing the heterodimer were pooled, dialyzed against 50 mM Tris-HCl pH 7.0, containing 25 mM NaCl, 1 mM EDTA, 1 mM dithiothreitol (DTT) and 10% glycerol, concentrated in Centriprep-30 and Centricon-30 to <0.5 ml, and stored at −20 °C. Enzymes were quantified by active site titration^64^,^65^ before biochemical studies.

### Mutagenesis

Site-directed mutagenesis was carried out by following the standard QuikChange^TM^ (Stratagene) protocol. The pT5m plasmid encoding the large subunit of the HIV-2_ROD_ RT was used as template^63^. Complementary mutagenic primers were used to amplify the entire pT5m(RODRT) plasmid in a thermocycling reaction carried out with high-fidelity Pfu DNA polymerase. The mutagenic primers used were: 5′-CATTTGCAATCAAGAGAAAGGACAAAAAC-3′ and 5′-GTTTTTGTCCTTTCTCTTGATTGCAAAT-3′ for K65R, 5′-TAAAGTCTTGCCAATGGGATGGAAGGGA-3′ and 5′-TCCCTTCCATCCCATTGGCAAGACTTTA-3′ for Q151M, and 5′-ATTATCATTCAGTACGTGGATGATATCT-3′ and 5′-AGATATCATCCACGTACTGAATGATAAT-3′ for M184V. Mutations found in the triple mutant K65R/Q151M/M184V were orderly added starting from mutant K65R, and then introducing mutation Q151M, and finally M184V. All constructs were verified by DNA sequencing.

### Pre-steady-state kinetic assays

Transient kinetics experiments were used to determine the catalytic constants *k*_pol_ and *K*_d_ for the incorporation of correct and incorrect nucleotides. Assays were carried out as previously described^44^,^66^,^67^, in 50 mM Tris/HCl buffer (pH 8.0), containing 50 mM KCl and 12 mM MgCl_2_ (for correct dNTPs) or 15 mM (for incorrect dNTPs), and varying concentrations of dNTP, using the heteropolymeric template-primer 31T/21P at 100 nM (Fig. 1). An excess of Mg^2+^ concentration over dNTP was used in all assays. The experiments were performed manually or with a rapid quench flow instrument (model QFM-400, Bio-Logic Science Instruments, Claix, France), upgraded with a mixer cross and a special mixer (Bio-Logic) that facilitated the determination of catalytic rate constants greater than 20 s^−1^. Oligonucleotides 21P and 31T (sequences provided in Fig. 1) were used as primer and template, respectively. Three additional primers (21PT, 21PG and 21PA) were used in mispair extension fidelity assays. These oligonucleotides differed from 21P in having T, G or A (instead of C) at their 3′-end. Before annealing to the template, primers were labelled at their 5′ terminus with [γ-^32^P]ATP (Perkin Elmer, Boston, MA, USA) and T4 polynucleotide kinase (New England Biolabs, Ipswich, MA, USA).

In misincorporation fidelity assays, dTTP incorporation reactions were performed under single-turnover conditions with 75–125 nM (active sites) RT and 100 nM template-primer. At appropriate time-points, the reactions were quenched with EDTA (0.3 M final concentration). The products were analysed by denaturing PAGE and quantified by phosphorimaging. Reactions involving the incorporation of incorrect nucleotides or mispair extension kinetics (i.e. dCTP, dGTP or dATP incorporation on 31T/21P, or the extension of G:T, G:G and G:A mispairs) were carried out with 120 nM RT and 100 nM template-primer. These conditions were chosen to eliminate the influence of the enzyme turnover rate (*k*_ss_), which interferes with the determination of low incorporation rates. Due to their lower catalytic rates, incorrect nucleotide incorporation assays and G:G and G:A mispair extension assays were carried out manually, as well as the longer incubation times needed for the determination of kinetic parameters in G:T mispair extensions. The kinetic constants *K*_d_ and *k*_pol_ were determined as described^67^, from curve-fitting using SigmaPlot, after calculating catalytic rates (*k*_obs_) at different dNTP concentrations using the burst equation: [P] = A × [1 − exp(−*k*_obs_ × *t*)] + *k*_ss_ × *t*, where [P] is the product concentration, A the amplitude of the burst, and *k*_obs_ the apparent kinetic constant of formation of the phosphodiester bond.

### M13mp2 *lacZα* forward mutation assays

The fidelity of DNA-dependent DNA synthesis was determined using the forward mutation assay^45^, under the previously described conditions^40^,^68^,^69^. Gap filling synthesis reactions were carried out at 37 °C during 60 min, in 25 μl of 25 mM Tris-HCl (pH 8.0) buffer containing 100 mM KCl, 2 mM DTT, 4 mM MgCl_2_, 250 μM of each dNTP and 100 nM RT. Each reaction contained 100–150 ng of gapped DNA. The gapped DNA was preincubated at 60 °C during 5 min to reduce secondary structure in the single-stranded DNA and avoid undesirable hybridizations. Nine or ten gap filling reactions were performed for each tested RT and the obtained products were analysed on a 0.8% agarose gel to check if the reactions had been completed. Reaction products were electroporated into *E. coli* MC1061 cells, and the transformed cells were plated onto M9 medium-containing plates with X-Gal and IPTG with CSH50 lawn cells^45^. Mutant frequencies were calculated as the ratio of mutant (light blue or colourless) plaques to the total number of plaques screened. Mutant phenotypes were confirmed by nucleotide sequencing of the phage replicative-form DNA using primer 5′-GCTTGCTGCAACTCTCTCAG-3′^69^. It should be noted that silent mutations and mutations that do not affect the β-galactosidase activity may not be detected in these assays. Error rates were calculated as described by Bebenek and Kunkel^45^. It has been estimated that this limitation results in a 2- to 3-fold underestimation of the actual mutation rates^70^.

## Additional Information

**How to cite this article:** Álvarez, M. *et al*. Fidelity of classwide-resistant HIV-2 reverse transcriptase and differential contribution of K65R to the accuracy of HIV-1 and HIV-2 reverse transcriptases. *Sci. Rep.* **7**, 44834; doi: 10.1038/srep44834 (2017).

**Publisher's note:** Springer Nature remains neutral with regard to jurisdictional claims in published maps and institutional affiliations.

## Supplementary Material

Supplementary Information

## Figures and Tables

**Table 1:** Pre-steady-state kinetic constants for the incorporation of dTTP on a heteropolymeric template-primer by WT and mutant HIV-2 RTs.

Enzyme	*k*_pol_ (s^−1^)	*K*_d_ (μM)	*k*_pol_/*K*_d_ (μM^−1^·s^−1^)
WT HIV-2 RT	13.6 ± 1.6	42.2 ± 11.1	0.32 ± 0.09
K65R HIV-2 RT	16.0 ± 1.1	53.7 ± 9.1	0.30 ± 0.06
Q151M HIV-2 RT	50.8 ± 4.7	99.0 ± 21.2	0.51 ± 0.12
M184V HIV-2 RT	27.5 ± 5.9	164 ± 77	0.168 ± 0.087
K65R/Q151M/M184V HIV-2 RT	19.1 ± 2.4	210 ± 53	0.091 ± 0.025
WT BH10 HIV-1 RT	11.6 ± 0.5	13.4 ± 1.9	0.86 ± 0.13

The template-primer 31T/21P was used as the substrate. Mean values ± standard deviations are given for each enzyme. Assays were performed independently at least three times. Reported values for the WT BH10 HIV-1 RT were taken from Matamoros *et al*.^44^.

**Table 2:** Pre-steady-state kinetic parameters for misincorporation.

Enzyme	Nucleotide	*k*_pol_ (s^−1^)	*K*_d_ (μM)	*k*_pol_/*K*_d_ (μM^−1^·s^−1^)	Misinsertion ratio (*f*_*ins*_)^a^
WT HIV-2 RT	dCTP	(3.4 ± 0.4) × 10^−2^	6597 ± 1508	(5.2 ± 1.3) × 10^−6^	(1.6 ± 0.6) × 10^−5^
	dGTP	(5.3 ± 0.4) × 10^−2^	2702 ± 654	(2.0 ± 0.5) × 10^−5^	(6.1 ± 2.4) × 10^−5^
	dATP	(4.0 ± 0.4) × 10^−3^	2715 ± 753	(1.5 ± 0.4) × 10^−6^	(4.6 ± 1.9) × 10^−6^
K65R HIV-2 RT	dCTP	(8.0 ± 0.6) × 10^−3^	3562 ± 783	(2.2 ± 0.5) × 10^−6^	(7.5 ± 2.2) × 10^−6^ (2.1)
	dGTP	(2.8 ± 0.2) × 10^−2^	1928 ± 542	(1.5 ± 0.4) × 10^−5^	(4.8 ± 1.7) × 10^−5^ (1.3)
	dATP	(1.0 ± 0.1) × 10^−3^	2980 ± 699	(3.4 ± 0.8) × 10^−7^	(1.1 ± 0.3) × 10^−6^ (4.1)
K65R/Q151M/M184V HIV-2 RT	dCTP	(7.3 ± 0.6) × 10^−3^	4985 ± 927	(1.5 ± 0.3) × 10^−6^	(1.6 ± 0.5) × 10^−5^ (1.0)
	dGTP	(2.3 ± 0.2) × 10^−2^	2400 ± 511	(9.6 ± 2.1) × 10^−6^	(1.1 ± 0.4) × 10^−4^ (0.6)
	dATP	(1.1 ± 0.1) × 10^−3^	1754 ± 277	(6.5 ± 1.1) × 10^−7^	(7.1 ± 2.3) × 10^−6^ (0.6)

The template-primer 31T/21P was used as the substrate. Data shown are the mean values ± standard deviation. Each of the assays was performed independently at least three times.

^a^*f*_ins_ = [*k*_pol_(incorrect)/*K*_d_(incorrect)]/[*k*_pol_(correct)/*K*_d_(correct)], where incorrect nucleotides were dCTP, dGTP or dATP, and the correct nucleotide was dTTP. Numbers between parentheses represent the relative increase of fidelity, as determined for each incorrect nucleotide as the ratio: *f*_ins_ (WT_HIV-2 RT)/*f*_ins_ (mutant RT).

**Table 3:** Pre-steady-state kinetic parameters for mispair extension.

Enzyme	Base pair at the 3′ end^a^	*k*_pol_ (s^−1^)	*K*_d_ (μM)	*k*_pol_/*K*_d_ (μM^−1^•s^−1^)	Mismatch extensión ratio (*f*_*ext*_)^b^
WT HIV-2 RT	G:T	2.41^c^	>8000	<2.4 × 10^−4^	nd^d^
	G:G	0.12 ± 0.01	2023 ± 381	(5.8 ± 1.2) × 10^−5^	(1.8 ± 0.6) × 10^−4^
	G:A	(3.1 ± 0.2) × 10^−3^	5885 ± 990	(5.3 ± 1.0) × 10^−7^	(1.6 ± 0.6) × 10^−6^
K65R HIV-2 RT	G:T	0.27^c^	>8000	<2.7 × 10^−5^	nd
	G:G	(1.5 ± 0.1) × 10^−2^	2758 ± 492	(5.4 ± 1.0) × 10^−6^	(1.8 ± 0.5) × 10^−5^ (9.8)
	G:A	<4.1 × 10^−4^	>8000	nd	nd
K65R/Q151M/M184V HIV-2 RT	G:T	0.27 ± 0.01	1299 ± 175	(2.1 ± 0.3) × 10^−4^	(2.3 ± 0.7) × 10^−3^ (0.3)
	G:G	(3.0 ± 0.3) × 10^−2^	2542 ± 773	(1.2 ± 0.4) × 10^−5^	(1.3 ± 0.5) × 10^−4^ (1.4)
	G:A	(1.5 ± 0.1) × 10^−3^	2213 ± 302	(6.8 ± 1.0) × 10^−7^	(7.4 ± 2.3) × 10^−6^ (0.3)

The template-primer 31T/21P was used as the substrate. Data shown are the mean values ± standard deviation. Each of the assays was performed independently at least three times.

^a^The first base corresponds to the template and the second to the primer.

^b^*f*_ext_ = [*k*_pol_(mismatched)/*K*_d_(mismatched)]/[*k*_pol_(matched)/*K*_d_(matched)]. Numbers between parentheses represent the relative increase of fidelity, as determined for each mispair as the ratio: *f*_ext_ (WT_HIV-2 RT)/*f*_ext_ (mutant RT).

^c^Indicated values are observed catalytic rates (*k*_obs_) at 10 mM concentration of dTTP.

^d^nd, not determined.

**Table 4:** Accuracy of WT and mutant RTs in M13mp2 *lac*Z*α* forward mutation assays.

RT	Total plaques	Mutant plaques	Mutant frequency^a^
WT HIV-2	9660	120	0.01242
K65R HIV-2	4750	56	0.01179 (1.1)
K65R/Q151M/M184V HIV-2	5142	53	0.01031 (1.2)
BH10 HIV-1	2915	58	0.01990 (0.6)

^a^Background frequencies in these assays were estimated to be less than one in 20,000 plaques^40^. Numbers between parentheses indicate the fold-increase in fidelity relative to the WT HIV-2 RT.

**Table 5:** Summary of error rates for WT and mutant RTs, for various classes of mutations, based on M13mp2 DNA-dependent DNA synthesis reactions.

Mutation type	WT HIV-2 (ROD)		K65R HIV-2 RT		K65R/Q151M/M184V HIV-2 RT		WT HIV-1 (BH10)	
	Number of errors	Error rate	Number of errors	Error rate	Number of errors	Error rate	Number of errors	Error rate
All classes	137	1/10027	57	1/11850	54	1/13541	63	1/6580
Base substitutions	88	1/8233	28	1/12723	24	1/16069	54	1/4049
Transitions	21 (24%)		1 (4%)		5 (21%)		14 (26%)	
Transversions	67 (76%)		27 (96%)		19 (79%)		40 (74%)	
Frameshifts	49	1/17506	29	1/14545	30	1/15220	9	1/28761
Insertions	0 (0%)		0 (0%)		1 (3%)		0 (0%)	
Deletions	49 (100%)		29 (100%)		29 (97%)		9 (100%)	
At runs^a^	5	1/91577	2	1/112575	1	1/243731	2	1/69086
At non-runs	44	1/9089	27	1/7283	29	1/7341	7	1/17240

^a^A run is here defined as a row of three or more identical nucleotides.
