# Supplementary material for: Fidelity of classwide-resistant HIV-2 reverse transcriptase and differential contribution of K65R to the accuracy of HIV-1 and HIV-2 reverse transcriptases
Source: Sci Rep. 2017 Mar 23;7:44834. doi: 10.1038/srep44834 (PMC5363063; doi:10.1038/srep44834)
Supplement: Supplementary Information [file srep44834-s1.pdf]

Supplementary information

Fidelity of classwide-resistant HIV-2 reverse transcriptase and differential contribution of K65R to the accuracy of HIV-1 and HIV-2 reverse transcriptases

Mar Álvarez, Alba Sebastián-Martín, Guillermo García-Marquina & Luis Menéndez-Arias

Centro de Biología Molecular "Severo Ochoa" (Consejo Superior de Investigaciones Científicas and Universidad Autónoma de Madrid), Madrid, Spain

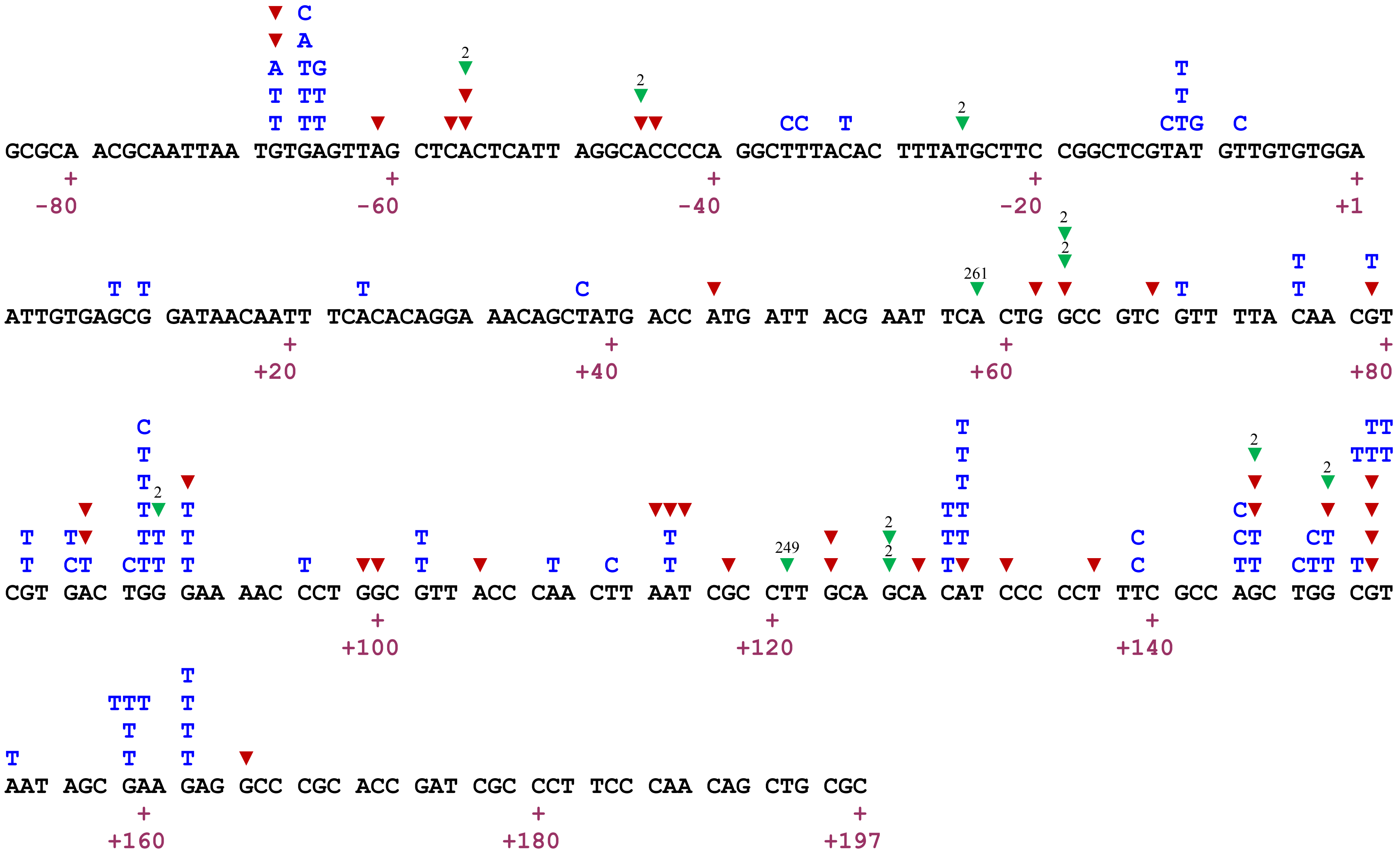

Supplementary Figure S1. Spectrum of mutations induced by the WT HIV-2$_{ROD}$ RT. Single-nucleotide substitutions are indicated by the letter corresponding to the new base (in blue) above the template sequence of the *lacZα* target. Inverted triangles indicate deletions of one nucleotide (red) or two or more nucleotides (green). On this last case, triangle is positioned in the 3´ end of the deletion and the number of deleted nucleotides is indicated above.

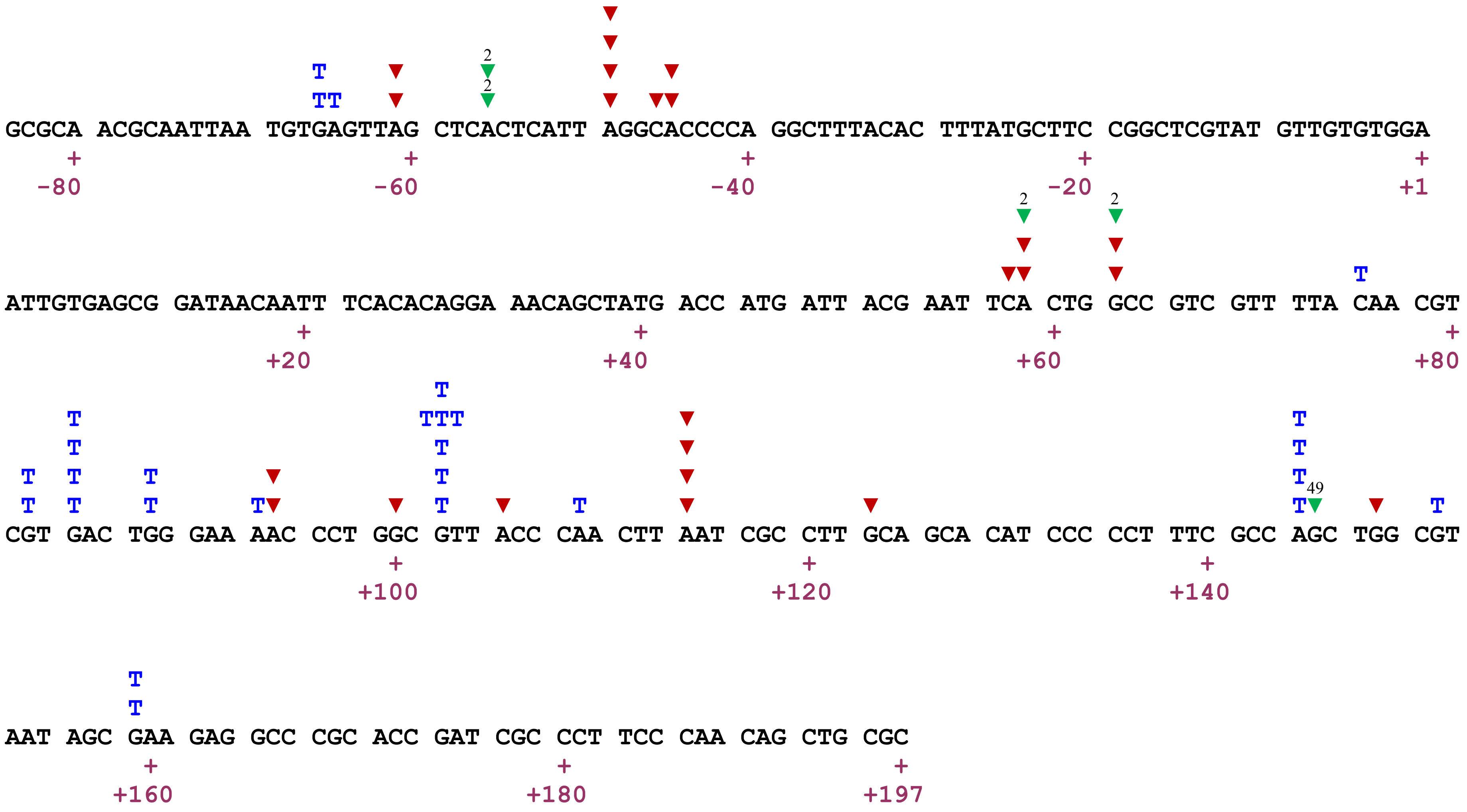

Supplementary Figure S2. Spectrum of mutations induced by HIV-2 RT mutant K65R. Single-nucleotide substitutions are indicated by the letter corresponding to the new base (in blue) above the template sequence of the *lacZα* target. Inverted triangles indicate deletions of one nucleotide (red) or two or more nucleotides (green). On this last case, triangle is positioned in the 3´ end of the deletion and the number of deleted nucleotides is indicated above.

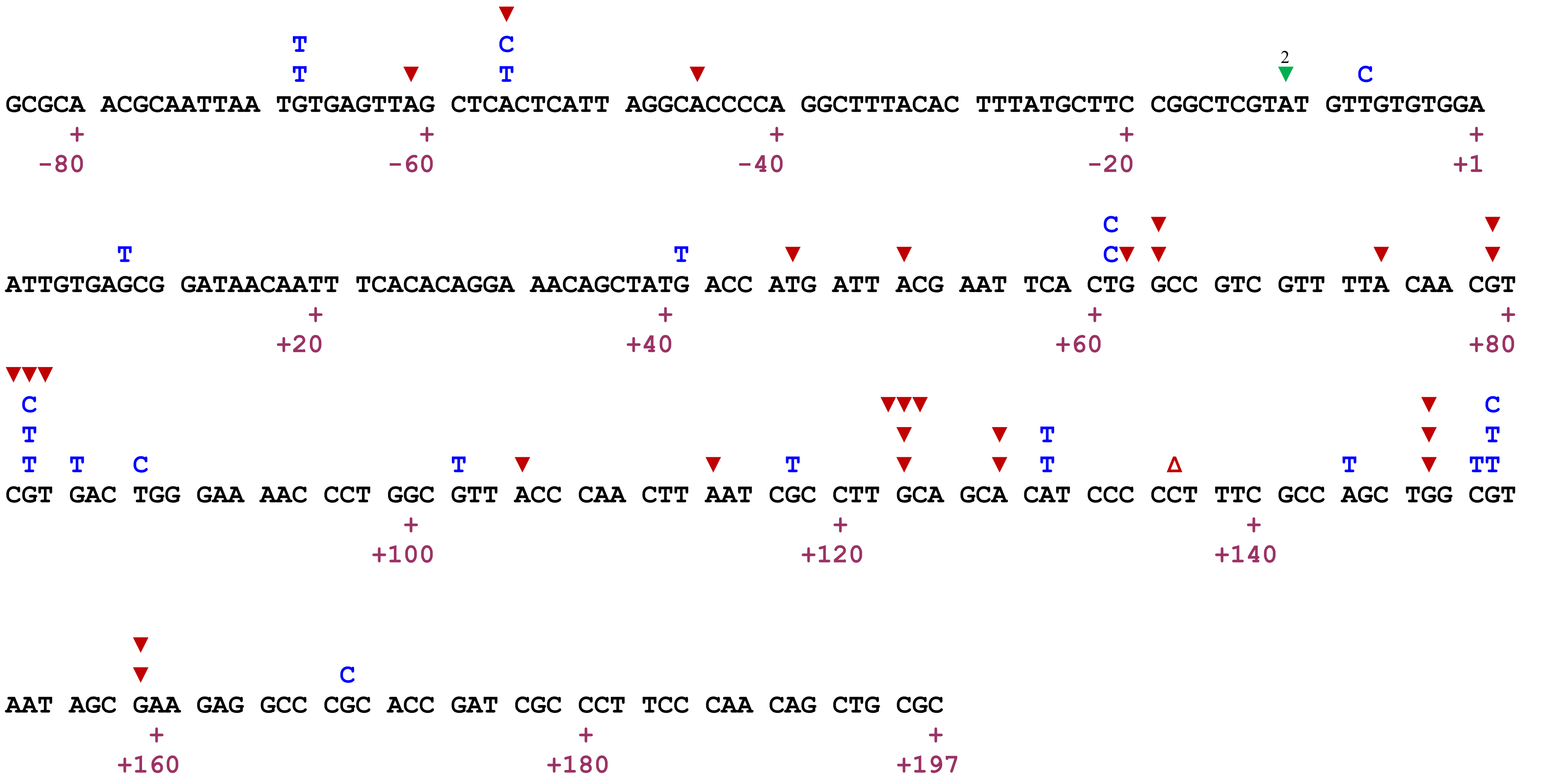

Supplementary Figure S3. Spectrum of mutations induced by HIV-2 RT mutant K65R/Q151M/M184V. Single-nucleotide substitutions are indicated by the letter corresponding to the new base (in blue) above the template sequence of the *lacZα* target. Open upright red triangles represent insertions of one nucleotide (duplication of the base where the triangle is positioned). Inverted triangles indicate deletions of one nucleotide (red) or two or more nucleotides (green). On this last case, triangle is positioned in the 3′ end of the deletion and the number of deleted nucleotides is indicated above.

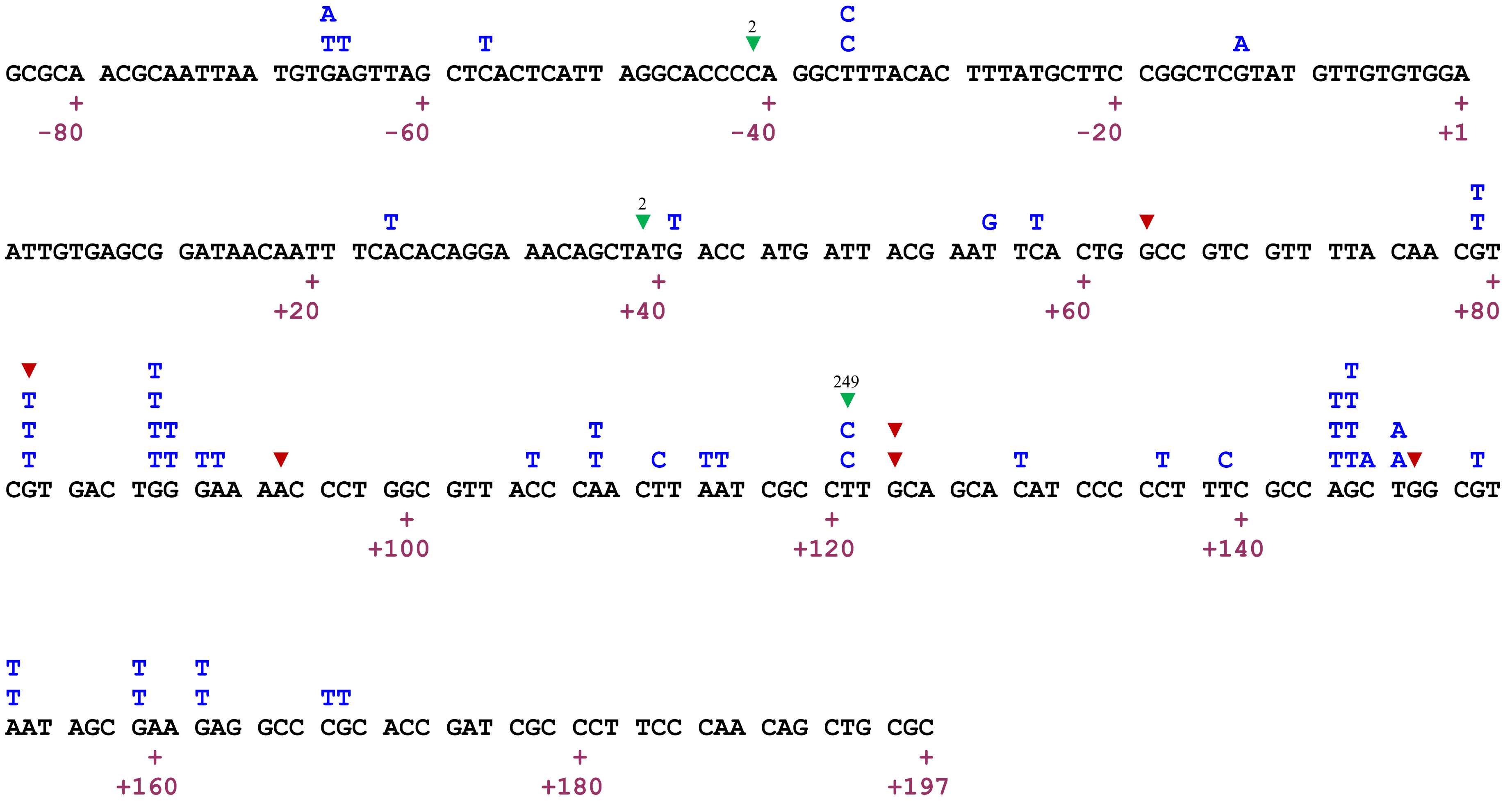

Supplementary Figure S4. Spectrum of mutations induced by WT HIV-1$_{BH10}$ RT. Single-nucleotide substitutions are indicated by the letter corresponding to the new base (in blue) above the template sequence of the *lac*Zα target. Inverted triangles indicate deletions of one nucleotide (red) or two or more nucleotides (green). On this last case, triangle is positioned in the 3´ end of the deletion and the number of deleted nucleotides is indicated above.
